# Feasible stenting of a large septal perforator providing a sizeable coronary collaterals

**DOI:** 10.21542/gcsp.2022.10

**Published:** 2022-06-30

**Authors:** Hussam Al Hennawi, Mohammad F. Mathbout, Abdulrahman Taftafa, Christopher D. Nielsen

**Affiliations:** 1 Department of Internal Medicine, Jefferson Abington Hospital, Abington, PA, USA; 2 Alfaisal University, College of Medicine, Riyadh, Saudi Arabia; 3 Medical University of South Carolina, Department of Cardiology, Charleston, South Carolina, USA

## Abstract

While coronary artery disease involving the septal perforator branches presents similar to diseases of major coronary arteries, management can present a challenge. Owing to their relatively small size, performing interventional procedures is often impractical in terms of selecting appropriate devices. Although larger septal perforator branches have been managed percutaneously, similar to major vessels, long-term sequelae and clinical effectiveness have been indeterminate. We present our experience in managing a patient with a stenosed septal perforator branch and challenging comorbidities.

## Background

Obstructive coronary artery disease (CAD) involving the septal perforator branches (SPBs) stemming from the left anterior descending artery (LAD) has been associated with CAD affecting major epicardial vessels. Serious complications including myocardial ischemia, conduction irregularities, and fatal arrhythmias may result following large SPB occlusion. The inherent characteristics of SPBs may present a challenge in performing revascularization procedures employing percutaneous coronary intervention (PCI), especially when the affected SPB has <2 mm caliber, distal location, and sharp stemming angulation rendering PCI device sizing ineffective given these sites are surgically inaccessible. Different PCI intervention techniques utilizing either stenting or no stenting approach have been described; nevertheless, evidence on clinical application, long-term effectiveness, and clinical outcomes is still lacking. We shed light on our experience in performing a feasible first SPB stenting that is deemed favorable in a post-bypass graft high-risk patient.

## Case Presentation

A 67-year-old male with a history of hypertension, coronary artery disease status post-2-vessel coronary artery bypass grafting in 2014, heart failure with reduced ejection fraction of 42%, atrial fibrillation on Eliquis, cerebrovascular accident status post-thrombectomy referred from an outside hospital with pneumonia and non-ST-segment-elevation myocardial infarction (NSTEMI).

**Figure 1.**
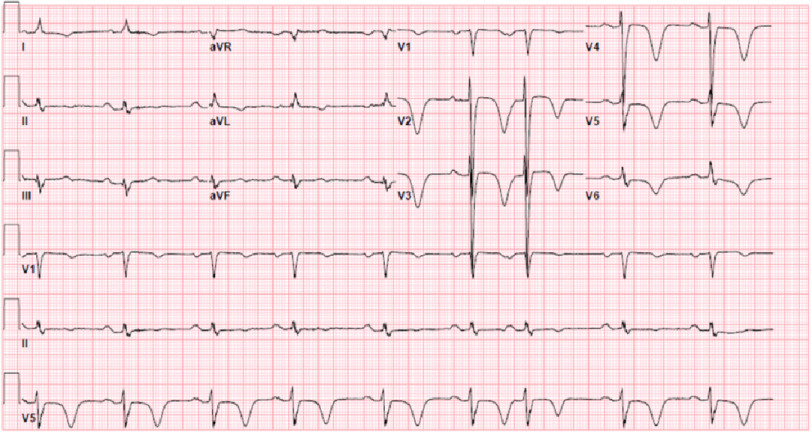
Electrocardiogram showing ST-segment depression with deep T-wave inversions predominantly in the anteroseptal leads.

At first, the patient complained of shortness of breath and reported worsening painful abdominal distension, at which point he sought medical attention. Initial investigations demonstrated elevated BNP levels. Chest computed tomography (CT) was negative for pulmonary embolism (PE), but showed right-sided pleural effusion and mild perihilar and peripheral opacities, likely representing the development of multifocal pneumonia. The patient received intravenous Lasix, antibiotics, and steroids. A 12-lead electrocardiogram (ECG) showed ST-segment depression with deep T wave inversions predominantly in the anteroseptal leads, raising suspicion of underlying myocardial ischemia (Figure 1).

**Figure 2.**
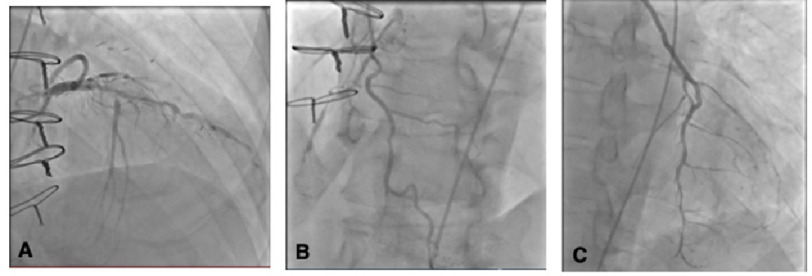
Coronary images. (A) High-grade LAD disease in the proximal and mid-vessel segments and very large first septal perforator branch with high-grade ostial stenosis. (B) Diffuse RCA disease, occluded mid-vessel, distal vessel fills *via* collaterals from first septal perforator branch. (C) Patent LIMA supplying mid-distal LAD.

His troponin I level was 918 ng/L (normal, ≤27 ng/L). The patient was subsequently transferred to the catheterization lab for further management. Coronary angiogram (CA) revealed a long segment of high-grade disease in the proximal and mid-distal left anterior descending artery (LAD), very large first septal perforator branch (SPB) with high-grade ostial stenosis (Figure 2A)—thought to be the culprit—and competitive filling of distal LAD from the left internal mammary artery (LIMA). The right coronary artery (RCA) showed diffuse disease with an occluded mid-vessel, and distal filling *via* collaterals from the first SPB (Figure 2B). LIMA to LAD was widely patent with diffuse mild disease in the native LAD (Figure 2C).

**Figure 3.**
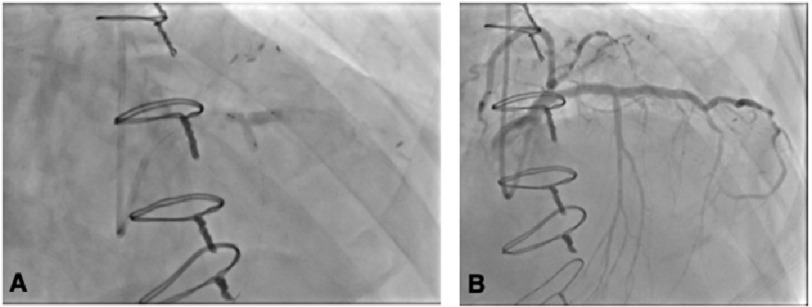
Coronary angiograms (right anterior oblique views) images. (A) Kissing balloon dilation was performed in the LAD and first SPB with 0% residual stenosis in the LAD and minimal residual stenosis at the ostium of the first SPB. (B) Successful stenting of the LAD and first SPB.

The LAD was stented with a 3.0 x 38 mm Resolute Onyx (drug-eluting) stent and was post-dilated with a 3.5 mm non-compliant balloon, yielding an excellent result and 0% residual stenosis. High-grade ostial stenosis of the first SPB branch was wired using a SuperCross^®^ (90° ) microcatheter, and the ostium was ballooned through the stent struts. High-grade stenosis remained, so the first SPB was stented with a 2.5 x 8 mm Resolute Onyx^®^ drug-eluting stent (DES), and kissing balloon dilation was performed in the LAD and first SPB. Excellent angiographic results were observed in the LAD and the first SPB, with 0% residual stenosis in the LAD and minimal residual stenosis at the ostium of the first SPB (Figure 3A, Figure 3B).

The patient performed well postoperatively. He was discharged on aspirin and Plavix in addition to Eliquis triple therapy for one month, followed by Plavix/Eliquis indefinitely.

## Discussion

The first SPB derives its unique anatomical importance as it delivers blood to the atrioventricular node and bundle of His in approximately 50% of the population^1^. The first SPB of the LAD is characteristically a large vessel with an approximate caliber similar to the diagonal artery, if not larger. Notably, SPBs contribute collateral circulation to already occluded coronary vessels, rendering the selected group of patients amenable to revascularization intervention.

Owing to a lack of evidence, PCI has rarely been performed in patients either because of questionable outcomes or because of feared complications as SPBs enter the heart at almost acute angles and course intramyocardially. Associated SPB stenting risks may include myocardial and coronary artery rupture, restenosis, and stent fracture due to the small stent size.

On the contrary, distinctive SPB features, including relatively large caliber (>2 mm) and wide branching takeoff angle, can accommodate stenting device delivery, resulting in successful revascularization even in patients with previous bypass^1^. Different modalities have been associated with successful revascularization including plain old balloon angioplasty (POBA), PCI with stenting, aspiration thrombectomy, and medical management only.

The revascularization of substantial SPB stenosis using POBA has been described in several studies^1–7^. Reported by the largest sample-size study involving 21 patients, Vemuri et al. associated significant large SPB POBA with a 95% success rate (a number similar to that of major epicardial coronary arteries)^1^. Nevertheless, other investigators reported acute vessel occlusion and complete heart block following this approach at an earlier or a later stage^3,4^. Since SPB obstructive lesions tend to be ostial, revascularization utilizing POBA may carry the risk of restenosis secondary to elastic recoil^5^. To overcome this countereffect, Cohen et al. described a rational atherectomy technique that can facilitate angioplasty primarily by debulking the atheromatous plaque, which exerts an anti-recoil effect^6^. However, this technique should target only large caliber SPBs as there might be a risk of slow flow or no reflow.

Revascularization through PCI and stenting can be challenging. Factors that may hinder successful intervention include vessel size, branch-ostial location, acute angle takeoff, and the intramyocardial course SPBs can harbor. Successful PCI with SPB stenting has been achieved in several reported cases, highlighting the significance of this approach. Deployment of a 2-mm stent achieved TIMI III flow in a 61-year-old male patient with CA showing a high-grade 2nd SPB lesion in the mid-vessel^7^. A successful 3-mm stent was deployed in a 42-year-old male patient post CABG presenting with unstable angina, whereby CA showed a 2.5- to 3-mm first SPB substantial tandem lesion extending to the body of the LAD, which was occluded distal to the origin of the first SPB^8^.

In our case, the first SPB stenting was favorable, given the large relative size and sizable collaterals supplying the chronically occluded RCA. There was no difficulty in overcoming the lesion or deploying the stent, yielding satisfactory results. Moreover, given the ostial origin, the lesion extended back into the body of the LAD; therefore, we had to utilize a 2.5 x 8 mm Resolute Onyx^®^ drug-eluting stent (DES), and kissing balloon dilation was performed in the LAD and first SPB. Secondly, angiography demonstrated patent LIMA supplying mid-distal LAD with filling of the distal LAD from LIMA. However, with the observed high-grade LAD disease in the proximal and mid-vessel segments, we proceeded with the decision to perform LAD stenting to enhance flow to the proximal diagonals that were not filling retrogradely.

Sole revascularization through aspiration thrombectomy has rarely been performed to manage atherosclerotic lesions of the SPBs. This approach was considered in a 78-year-old female who had chest pain and ST-segment elevation in leads V_1_ and V_2_ where CA showed acute thrombotic occlusion of a large-caliber first SPB^8^. Aspiration thrombectomy achieved 10–20% residual occlusion, TIMI III flow was achieved, and her EKG normalized.

Without justified invasive intervention, a medical management-only approach can be considered. This approach has been described in an asymptomatic 39-year-old male who presented with NSTEMI, 5800 ng/L troponin I level, and no discernable ischemic EKG changes^9^. CA suggested the culprit lesion involving occlusion of the first SPB. However, given the small caliber of the vessel, absence of symptoms, and no hemodynamic or electrical instability, clinical improvement was anticipated from medical therapy alone, whereby the patient was asymptomatic during his 2-month follow-up visit.

### What we learned?

• Given the lack of evidence supporting revascularization in SPBs, our experience demonstrates that PCI with stenting may be considered a feasible option for favorable outcomes in a selected group of patients.
• Prospective studies are needed to investigate long-term outcomes and address complications.
